# Incremental Costs and Cost Effectiveness of Intensive Treatment in Individuals with Type 2 Diabetes Detected by Screening in the ADDITION-UK Trial: An Update with Empirical Trial–Based Cost Data^☆^

**DOI:** 10.1016/j.jval.2017.05.018

**Published:** 2017-12

**Authors:** Michael Laxy, Edward C.F. Wilson, Clare E. Boothby, Simon J. Griffin

**Affiliations:** 1 Institute of Health Economics and Health Care Management, Helmholtz Zentrum München, Neuherberg, Germany; 2 German Center for Diabetes Research (DZD), Neuherberg, Germany; 3 Medical Research Council Epidemiology Unit, University of Cambridge School of Clinical Medicine, Cambridge, UK; 4 Cambridge Centre for Health Services Research, University of Cambridge School of Clinical Medicine, Cambridge, UK

**Keywords:** ADDITION trial, cost effectiveness, intensive treatment, screen-detected diabetes

## Abstract

**Background:**

There is uncertainty about the cost effectiveness of early intensive treatment versus routine care in individuals with type 2 diabetes detected by screening.

**Objectives:**

To derive a trial-informed estimate of the incremental costs of intensive treatment as delivered in the Anglo-Danish-Dutch Study of Intensive Treatment in People with Screen-Detected Diabetes in Primary Care-Europe (ADDITION) trial and to revisit the long-term cost-effectiveness analysis from the perspective of the UK National Health Service.

**Methods:**

We analyzed the electronic primary care records of a subsample of the ADDITION-Cambridge trial cohort (n = 173). Unit costs of used primary care services were taken from the published literature. Incremental annual costs of intensive treatment versus routine care in years 1 to 5 after diagnosis were calculated using multilevel generalized linear models. We revisited the long-term cost-utility analyses for the ADDITION-UK trial cohort and reported results for ADDITION-Cambridge using the UK Prospective Diabetes Study Outcomes Model and the trial-informed cost estimates according to a previously developed evaluation framework.

**Results:**

Incremental annual costs of intensive treatment over years 1 to 5 averaged £29.10 (standard error = £33.00) for consultations with general practitioners and nurses and £54.60 (standard error = £28.50) for metabolic and cardioprotective medication. For ADDITION-UK, over the 10-, 20-, and 30-year time horizon, adjusted incremental quality-adjusted life-years (QALYs) were 0.014, 0.043, and 0.048, and adjusted incremental costs were £1,021, £1,217, and £1,311, resulting in incremental cost-effectiveness ratios of £71,232/QALY, £28,444/QALY, and £27,549/QALY, respectively. Respective incremental cost-effectiveness ratios for ADDITION-Cambridge were slightly higher.

**Conclusions:**

The incremental costs of intensive treatment as delivered in the ADDITION-Cambridge trial were lower than expected. Given UK willingness-to-pay thresholds in patients with screen-detected diabetes, intensive treatment is of borderline cost effectiveness over a time horizon of 20 years and more.

## Introduction

Diabetes mellitus is an increasing public health problem, associated with costly micro- and macrovascular complications, reduced quality of life, and premature death [1], [2], [3], [4]. The direct and indirect societal costs of diabetes in the United Kingdom are expected to rise from £22 billion in 2010 to £35 billion in 2030, and a large share of this financial burden is attributable to the treatment of diabetic complications in patients with type 2 diabetes [5]. Cost-effective disease management strategies are therefore needed to diminish the burden of the disease on patients and health care systems.

Previous research has shown that intensive multifactorial treatment, including management of cardiovascular risk factors and glycemic control, reduces the risk of cardiovascular events and is an effective and cost-effective intervention for patients with long-standing diabetes [6], [7], [8]. There is also solid evidence that tight control of glucose and blood pressure in newly routinely diagnosed patients is an effective and cost-effective strategy [9], [10], [11]. Conversely, little is known about the cost-effectiveness of intensive treatment in individuals with type 2 diabetes detected by screening who, all else being equal, will typically be at an earlier stage in the disease.

The diagnosis of diabetes in routine care settings occurs on average a couple of years after physiological onset [12]. Because of improvements in quality of care and ongoing considerations about population-based screening, this lead time is expected to decrease, resulting in a large number of patients who could potentially benefit from early intensive treatment.

The pragmatic cluster-randomized Anglo-Danish-Dutch Study of Intensive Treatment in People with Screen-Detected Diabetes in Primary Care-Europe (ADDITION) trial studied the effect of intensive multifactorial treatment compared with routine care on cardiovascular morbidity and mortality in individuals with type 2 diabetes detected by screening [13], [14]. The results showed a nonstatistically significant relative risk reduction in the incidence of the composite cardiovascular end point over a time horizon of 5 years [15].

Of note, levels of cardiovascular risk factors improved modestly over the 5 years of the trial, and a modeling study using the UK Prospective Diabetes Study (UKPDS) risk equations indicated that the cardiovascular risk might be reduced in the long- term [15], [16]. Despite this, an initial cost-effectiveness analysis using the UKPDS outcomes model incorporating conservative protocol-driven intervention cost estimates showed that over a time horizon of 30 years, the intervention was not cost effective according to current UK willingness-to-pay (WTP) thresholds (incremental cost-effectiveness ratio [ICER] ~£37,500 per quality-adjusted life-year [QALY] vs. WTP thresholds of £20,000–£30,000) [17], albeit with substantial decision uncertainty (31% probability that the ICER is <£30,000). Nevertheless, as we know from a previous study that the adherence of general practitioners (GPs) to the trial protocol was not perfect, the incremental costs of the intervention actually delivered might have been lower than expected [18]. Therefore, although our outcomes assessment would be valid, we may have overestimated the incremental cost. Had the intensive treatment regimen been highly cost-effective or cost-ineffective and with a high degree of certainty, further exploration would have been of no value. Nevertheless, given the proximity of the ICER to the (upper) threshold and the level of decision uncertainty, we felt further investigation into the intervention costs was justified.

The objective of this study was therefore to estimate the incremental costs of early intensive treatment as delivered in ADDITION using empirical data from electronic primary care records. We then used this new information to update our previous estimate of the long-term (10–30 years) cost-effectiveness analysis of the ADDITION intervention in the United Kingdom from a National Health Service (NHS) perspective, in a manner consistent with an iterative approach to research and decision making [19], [20], [21].

## Methods

### Study Design and Study Population

The ADDITION-UK (NCT00237549) study was a part of the ADDITION-Europe study and consisted of two phases—a screening program and a pragmatic, cluster-randomized trial comparing the effect of early intensive treatment with that of routine care in individuals with type 2 diabetes detected by screening on a composite end point of cardiovascular morbidity and mortality [13], [14]. High-risk individuals without known diabetes aged 40 to 69 years registered in 69 primary care surgeries within a range of 100 miles of the study centers in Cambridge and Leicester were invited for stepwise screening. Of these, 867 individuals (from 49 surgeries) from Cambridge and 159 individuals (from 20 surgeries) from Leicester with type 2 diabetes detected by screening participated in the primary care–based intervention study (ADDITION-UK). Two participants withdrew before year 5, leaving a total study size of 1024 participants. Details of the study protocol including assessment of primary end points and inclusion and exclusion criteria have been published elsewhere [15]. The study was approved by local ethics committees, and all participants provided informed consent.

### Routine Care versus Intensive Treatment

Patients were treated according to the treatment allocation of their surgery. Patients in the routine care arm in Leicester and Cambridge received diabetes care through the UK NHS on the basis of contemporary UK treatment guidelines [22], [23], [24]. In the intensive treatment arm, additional features were added to routine care. Some of these intensive treatment features differed between the Leicester and the Cambridge GP surgeries.

In Leicester, intensive treatment was delivered by a specialist team of doctors, nurses, and dieticians within peripatetic community clinics according to the Diabetes Education and Self-Management Programme, which is a group education program delivered by two registered health care professionals in one 6-hour session [25]. The curriculum focuses on lifestyle changes and medication adherence using theories of efficient goal setting and self-efficacy. In addition, in the first year after diagnosis, patients were offered bimonthly appointments with a nurse or a GP in a community peripatetic clinic, and 4-monthly thereafter.

In Cambridge, primary care surgeries received funding for more frequent contacts between patients and practitioners. An initial practice-based academic detailing session conducted by a local diabetologist and an academic GP and interactive practice-based audit and feedback sessions were organized around 6 and 14 months after the initial education session and annually thereafter. Surgery staff received theory-based education materials to hand over to patients, and participants were encouraged to initiate lifestyle changes, to adhere to medication schemes, to self-monitor blood glucose levels if given a glucometer by their practice, and to attend annual health checks.

In addition, in all intensive treatment arm surgeries (Leicester and Cambridge), GPs were advised to follow treatment algorithms for medication with glucose-lowering, angiotensin-converting enzyme inhibiting, lipid-lowering, and platelet-inhibiting medication that were slightly tighter than those in contemporary UK treatment guidelines [13], [22], [23], [24]. According to the protocol, therapy with glucose-lowering medication was indicated for patients with a glycated hemoglobin A_1c_ (HbA_1c_) level of more than 6.5%, therapy with angiotensin-converting enzyme inhibitors was indicated for patients with blood pressure higher than 120/80 mm Hg or prevalent cardiovascular disease, statin therapy was indicated for patients with a cholesterol level of higher than 3.5 mmol/L, and aspirin therapy was indicated for all patients without specific contraindications [14].

### Incremental Costs of Intensive Treatment in ADDITION-Cambridge

#### Data source and operationalization

Because of the high cost of assessing and extracting data from electronic primary care records, it was decided in the planning phase of the study that only the records of a subset of the study would be assessed. Records of each participant with a primary end point (i.e., cardiovascular event) plus the records of two random participants from the same GP surgery without a primary end point within the 5-year trial period were accessed. Consequently, the records of 30 participants with a primary end point and of 60 participants without a primary end point from the intensive treatment arm and the records of 33 participants with a primary end point and of 66 participants without a primary end point from the routine care arm were accessed.

These records comprised information on consultations with outpatient health care professionals, prescribed medications, and diagnostic tests from the date of diagnosis (between 2002 and 2005) until the end of the year 2010 (~80,000 observations in total). Costs associated with the use of these services were obtained by multiplying the number of consumed resources by their respective unit prices. Unit prices for consultations with GPs and nurses were extracted from the Personal Social Services Research Unit report on unit costs of health and social care [26]. Prices for all other consultations were taken from the National Schedule of Reference Costs 2009–10 for NHS Trusts [27]. The Prescription Cost Analysis 2010 was used to assign unit prices for prescribed medications [28]. Because of incomplete or ambiguous information from the free-text records, no unit costs could be assigned to around 1% of the recorded used resources. These services were therefore priced according to the mean unit price of used units for the person and year.

On the basis of the study protocol, we allocated cost items to the following categories:

1. costs for consultations related to the trial protocol (contacts with GPs and nurses);
2. costs for medication related to the trial protocol (glucose-lowering drugs, blood pressure–lowering drugs, cholesterol-lowering drugs, and platelet-inhibiting drugs); and
3. costs for all other services (contacts with other primary health care professionals and outpatient specialists, other medications, and diagnostic tests).

#### Statistical analyses

In the long-term decision model, costs for primary care and medication accrue until a person dies. As input parameters for this decision model, one therefore needs an empirical estimate that describes the difference in average costs between a person alive in the intensive treatment arm and a person alive in the routine care arm. For this, we subdivided the 5-year analysis period into five annual intervals (year 1 to year 5 after diagnosis) and included the observation year in which a person died, but excluded subsequent years from the analysis. After exclusion of 16 participants for whom none or less than 1 year’s data were available, 173 participants (from 34 surgeries; mean cluster size = 5; minimum = 2, maximum = 17) provided 841 person-years of data until death. Medication data were missing for 18 of the 173. These costs were imputed with Markov chain Monte-Carlo procedures using model covariates and available annual cost values for consultations, medications, and diagnostic tests. This yielded a final analysis sample of 173 participants with 841 complete observation years.

Here, we first descriptively reported the resource utilization of categories 1 and 2, which has been described in detail elsewhere [18]. Second, we analyzed the annual incremental costs of intensive treatment for each resource utilization category separately using generalized linear models (GLMs). We tested a GLM with identity-link and Gaussian distribution (i.e., ordinary least-squares model), a GLM with log-link and gamma/Poisson distribution, and a GLM with square-root-link and gamma/Poisson distribution (in models with a log link, all zero costs were set to a nominal £1) [29]. Results from these models were very similar; for overall costs, we decided to use the ordinary least-squares model, which is the simplest and yielded the most conservative cost estimates [30]. Models accounted for observation years being clustered into patients and patients being clustered into primary care surgeries (three-level random intercept model) and were adjusted for age, sex, and HbA_1c_ level at diagnosis. We also introduced an interaction term between the year after diagnosis and the treatment status to capture potential trends over time. In a second step, using the same statistical methods, we estimated the total annual incremental costs. To account for the nonrandom selection of the analyzed subsample, we introduced a general weighting factor, representing the inverse probability of being included in this analysis, on the basis of the status of having a primary end point [31].

These models yielded mean estimates and standard errors (SEs) for the annual incremental costs of consultations (*β*_*c*onsultations-Cambridge_; SE_consultations-Cambridge_), medication (*β*_medication-Cambridge_; SE_medication-Cambridge_), and the intervention as a whole, including other primary care services (*β*_total-Cambridge_; SE_total-Cambridge_). Analyses were performed with SAS 9.3 using the GLIMMIX, MI, and MIANALYZE procedures (SAS Institute, Inc., Cary, NC).

### Long-Term Cost Effectiveness of Intensive Treatment in ADDITION-UK/Cambridge

The long-term cost effectiveness of ADDITION used the outputs from the UKPDS model as per the original model, with the updated short-term intervention costs from the electronic primary care records. The methods are briefly described here. The analysis is conducted from the perspective of the NHS. Because we have empirical data on the intervention costs only from the Cambridge centers but not from the Leicester centers, we first update the previous cost-effectiveness analyses for ADDITION-UK (Leicester and Cambridge). In a second step we report a separate long-term cost-effectiveness analysis for ADDITION-Cambridge only.

#### Quality-adjusted life-years

The UKPDS outcomes model v1.3 was applied to simulate the individual accumulated QALYs of patients [17], [32]. The UKPDS Outcomes Model is a widely used individual-level state transition simulation model (i.e., a microsimulation model) based on data from a UK population and applicable for the given evaluation context [33]. Its performance has been tested against the ADDITION 5-year outcomes in a previous study showing a moderate calibration and discrimination [34]. The model predicts future events (ischemic heart disease, myocardial infarction, heart failure, stroke, amputation, blindness, and renal failure) and death as a function of several values at diagnosis of diabetes (e.g., sex, ethnicity, and duration of diabetes) and on the basis of values of risk factors at diagnosis and in subsequent years (e.g., smoking, body mass index, cholesterol, high-density lipoprotein, HbA_1c_ level, and systolic blood pressure). Results on risk factor changes and effects on micro- and macrovascular events over the 5-year observation period have been reported previously and are summarized in Appendix 1 in Supplemental Materials found at doi:10.1016/j.jval.2017.05.018 [15], [16], [17], [35]. Utility decrements associated with the modeled events were obtained from the published literature, and the additive method was used for patients with multiple events (see Appendix 2 in Supplemental Materials found at doi:10.1016/j.jval.2017.05.018) [36], [37], [38].

#### Costs

We assumed that the costs for patients in the intensive treatment arm comprise the costs of treatment of complications plus the costs of delivering the intervention itself, including costs for planning and implementation and for extra consultations and medication, whereas in the routine care arm, only the costs of the treatment of complications occur. All costs were calculated in British pounds for the price year 2009/2010. The price year was chosen to maintain comparability with the previous economic analysis [17].

##### Treatment of events/complications

As for the effects, we used the UKPDS outcomes model v1.3 to estimate the per-patient costs for the treatment of events and complications [32]. Unit costs for the treatment of complications were obtained from the UKPDS study and other published literature (see Appendix 2 in Supplemental Materials) [37], [38], [39]. Again, the additive method was used to calculate costs in case of multiple complications or events.

##### Planning and implementation

The previously published internal accounting showed average per-patient costs of £375 in Cambridge and £71 in Leicester for the planning and implementation (teaching and feedback sessions) of the study [17]. These values were also used in this analysis.

##### Extra consultations and medication

For Cambridge (n = 867), we used the empirically derived cost estimates (*β*_consultations-Cambridge_, SE_consultations-Cambridge_, *β*_medication-Cambridge_, and SE_medication-Cambridge_). For Leicester (n = 159), no empirical cost data were available, and we used the cost estimates from the internal accounting [17], which were used for the protocol-based cost-effectiveness analysis [17]: *β*_consultations-Leicester_ (annual per-patient costs for extra consultations in years 1–5) = £880/5 = £176 and *β*_medication-Leicester_ (annual per-patient costs for extra medication in years 1–5 and thereafter) = £52.5. A detailed description of the protocol-based cost estimates is presented in Table 1.

**Table 1:** Protocol-based and empirical cost estimates (£) used in the initial [17] and updated base-case cost-effectiveness analyses

**Cost component**	**Center**	Per protocol*				**Trial-based**†			
		**Accumulated**		**Annually**		**Accumulated**		**Annually**	
		**Mean**	**SE**	**Mean**	**SE**	**Mean**	**SE**	**Mean**	**SE**
		*Upfront costs*							
Planning and implementation	Cambridge (n = 452)	375.1	–	–	–	375.1	0.0	–	–
	Leicester (n = 61)	71.2	–	–	–	71.2	0.0	–	–
		*Years 1–5*‡							
Extra consultations	Cambridge (n = 452)	311.3§	–	62.3||	–	145.5	161.5	29.1¶	32.3¶
	Leicester (n = 61)	880.1§	–	176.0||	–	880.1	976.9#	176.0	195.4#
Extra medication	Cambridge (n = 452)	262.5	–	52.5	–	273.0	142.5	54.6¶	28.5¶
	Leicester (n = 61)	262.5	–	52.5	–	262.5	137.0#	52.5	27.4#
		Year 6 until …							
		end of observation period				death or end of observation period			
Extra medication (years 6–10)	Cambridge (n = 452)	199.6**	–	52.5	–	183.7††	95.9	54.6¶	28.5¶
	Leicester (n = 61)	199.6**	–	52.5	–	203.4††	106.2#	52.5	27.4#
Extra medication (years 6–20)	Cambridge (n = 452)	509.1**	–	52.5	–	386.9††	201.9	54.6¶	28.5¶
	Leicester (n = 61)	509.1**	–	52.5	–	434.9††	227.0#	52.5	27.4#
Extra medication (years 6–30)	Cambridge (n = 452)	728.5**	–	52.5	–	444.3††	231.9	54.6¶	28.5¶
	Leicester (n = 61)	728.5**	–	52.5	–	520.3††	271.6#	52.5	27.4#

SE, standard error.

⁎ Protocol-based cost estimates according to the internal accounting of Tao et al. [17].

† Empirical cost estimates according to the analysis on a subsample of the ADDITION sample.

‡ Accumulated costs described without discounting.

§ Costs were assumed to occur from years 1 to 3.

|| Annual costs if distributed over 5 y.

¶ *ß*s and SEs extracted from Table 3.

# SEs in Leicester were assumed to be proportional to the ones in patients from Cambridge.

⁎⁎ Calculated using $\sum_{t=6}^{\mathrm{time}\;\mathrm{horizon}}\frac{\beta_{\;\mathrm{annual}\;\mathrm{medication}\;\mathrm{cost}}(t)}{{\left(1\;+\;0.035\right)}^{t}}$.

†† Calculated using $\sum_{t=6}^{\mathrm{LE}}\frac{\beta_{\;\mathrm{annual}\;\mathrm{medication}\;\mathrm{cost}}\left(t\right)}{{\left(1\;+\;0.035\right)}^{t}}$, where modeled life expectancy (LE) for the 10-, 20-, and 30-y time horizons averaged ~9.4, ~15.2, and ~17.0 y in Cambridge and ~9.6, ~16.3, and ~19.3 y in Leicester.

#### Statistical analysis

For patients in both trial arms, the individual 10-, 20-, and 30-year accumulated QALYs and costs for the treatment of complications were projected by running simulations with 1000 inner model loops and 100 bootstraps of the UKPDS outcomes model v.1.3 with a cycle length of 1 year [32]. Both costs and QALYs were discounted at a rate of 3.5% according to the guidelines of the National Institute for Health and Care Excellence (NICE) [40]. Some minor adjustments to the input data were performed before running the model: Patients with unknown or unclassifiable ethnicity were excluded from the analysis (n = 25), and values of atrial fibrillation, peripheral vascular disease, ischemic heart disease, congestive heart failure, amputation, blindness, and renal failure, which were not collected in ADDITION, were set to 0. Furthermore, missing values of input variables were imputed via Markov chain Monte-Carlo procedures (n = 5 imputations), and means and SEs were subsequently derived using Rubin’s rules (information on the missing data is provided in Appendix 3 in Supplemental Materials found at doi:10.1016/j.jval.2017.05.018).

As a base-case scenario, we calculated the incremental cost-effectiveness for ADDITION-UK, including patients from Cambridge and Leicester. To the simulated costs for the treatment of complications that occur in both treatment arms, for patients in the intervention arm, we added the per-patient mean costs for planning and implementation of the intervention, the discounted per-patient mean costs for extra consultations in years 1 to 5 $\left(\sum_{t=1}^{5}\frac{\beta_{\mathrm{consultations}}(t)}{{\left(1\;+\;0.35\right)}^{t}}\right)$ , and the discounted per-patient mean costs for medication until death $\left(\sum_{t=1}^{\mathrm{LE}}\frac{\beta_{\mathrm{medication}}\left(t\right)}{{\left(1\;+\;0.035\right)}^{t}}\right)\;$. Life expectancy (LE) for the 10-, 20-, and 30-year time horizons averaged approximately 9, 15, and 17 years, respectively. SEs of the different cost components were summed in an additive manner. In parallel to the method described by Tao et al. [17], the resulting means and SEs of QALYs and costs at patient level were used to conduct a bootstrap analysis (n = 500) adjusting for center, age at diagnosis, sex, and HbA_1c_ level at baseline.

We report the ICERs for the 10-, 20-, and 30-year time horizons and the probability of the intervention being cost effective given a WTP threshold of £30,000. We also illustrate the decision uncertainty with a scatterplot in the cost-effectiveness plane and a cost-effectiveness acceptability curve.

Analyses of modeled scenarios were conducted using the UKPDS outcomes v1.3 model and Microsoft Excel (Redmond, WA). The article was written according to the Consolidated Health Economic Evaluation Reporting Standards statement [41].

#### Sensitivity analysis

In additional analyses, we assumed that not only were the costs of medication incurred until death, but the total incremental primary care costs, including costs of consultations and other primary care services, were also incurred until death $\left(\sum_{t=1}^{\mathrm{LE}}\frac{\beta_{\mathrm{total}}\left(t\right)}{{\left(1\;+\;0.035\right)}^{t}}\right).$

One-way sensitivity analyses were performed on the main model for ADDITION-UK with varying treatment costs, utility decrements, and discount rates using the 30-year simulation data. The range for the discount rate (0%–5%) was guided by NICE guidelines suggesting a discount rate of 3.5% as base case and 1.5% in sensitivity analyses [42]. The range for utility decrements and unit costs (−20% to +20%) was guided by the coefficient of variation of parameter estimates that averaged approximately 8% to 12% in the data sources from which the input parameters were taken [36], [39].

We also adjusted our models to most recently available prices because relative prices, particularly for medications, might have changed substantially since 2010. The Personal Social Services Research Unit Costs of Health and Social Care 2010 and 2015 [43], [44], the British National Formulary for 2010 and 2016 [45], and the NHS trust reference cost schedules for 2009/2010 and 2015/2016 [46], [47] were used to retrieve relative price changes for GP and nurse contacts, for cardiometabolic medications, and for hospitalizations of diabetes-related complications (see Appendix 4 in Supplemental Materials found at doi:10.1016/j.jval.2017.05.018).

## Results

### Study Design and Study Population

Baseline characteristics of the total UK sample and of the weighted Cambridge subsample are presented in Table 2. The mean age of the UK sample was around 61.5 years. No substantial differences were observed between the UK sample (n = 1024) and the weighted Cambridge subsample from which empirical cost data were available (n = 173).

**Table 2:** Baseline characteristics of the ADDITION population trial cohort

**Characteristic**	ADDITION-UK (Cambridge + Leicester)*		**ADDITION-Cambridge analysis subsample (weighted**†**)**	
	**IT (n = 513)**	**RC (n = 511)**	**IT (n = 82)**	**RC (n = 91)**
Primary endpoint during follow-up period (%)	7.2	7.5	6.8	7.7
Sex, female (%)	36.6	40.7	40.8	39.4
Age (y), mean ± SD	61.1 ± 7.2	60.1 ± 7.5	61.8 ± 7.3	61 ± 7.1
BMI, mean ± SD (kg/m^2^)	33.1 ± 5.6	33.0 ± 5.9	33.4 ± 5.2	34 ± 5.7
Total cholesterol (mmol/L), mean ± SD	5.3 ± 1.1	5.5 ± 1.2	5.4 ± 1.1	5.6 ± 1.2
HDL (mmol/L), mean ± SD	1.17 ± 0.4	1.2 ± 0.3	1.2 ± 0.3	1.2 ± 0.3
Systolic blood pressure (mm Hg), mean ± SD	142.0 ± 20.1	143.1 ± 19.4	141.6 ± 21	142.5 ± 20.6
HbA_1c_ (%), mean ± SD	7.3 ± 1.7	7.3 ± 1.7	7.7 ± 2.2	7.4 ± 1.7

BMI, body mass index; HbA_1c_, glycated hemoglobin; HDL, high-density lipoprotein; IT, intensive treatment; RC, routine care.

⁎ Of the total sample (1026), 2 withdrew from the study.

† Weighting factor: inverse probability of being included in the study on the basis of the status of having a primary end point.

### Incremental Costs in ADDITION-Cambridge

**Fig. 1:**
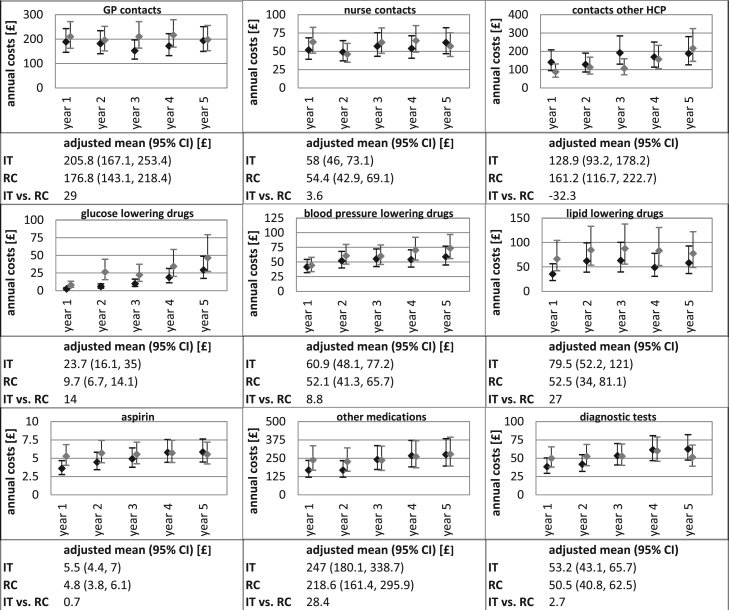
Adjusted means of annual primary care costs according to IT (gray) and RC (black) in years 1–5 (generalized linear model with a gamma distribution and log link with a main effect for the intervention and for time since diagnosis and an interaction term between intervention and time; adjusted for sex and age of diagnosis and baseline HbA_1c_; accounted for patients being clustered in GP surgeries and observations clustered in patients) no SE is available for the cost difference. CI, confidence interval; GP, general practitioner; HbA_1c_, glycated hemoglobin A_1c_; HCP, health care professional; IT, intensive treatment; RC, routine care; SE, standard error.

The primary care cost components for patients in the intensive treatment and routine care arm are illustrated in Figure 1. Respective resource utilization for contact with health care professionals and medication related to the trial protocol is illustrated in Appendix 5 in Supplemental Materials found at doi:10.1016/j.jval.2017.05.018. Most costs are attributable to contacts with GPs, metabolic and cardioprotective medication, and other types of medications. The annual costs for contacts with GPs, nurses, and other health care professionals and the annual costs for glucose-lowering drugs, blood pressure–lowering drugs, lipid-lowering drugs, aspirin, other medication, and diagnostic tests stayed constant or increased over the 5-year time horizon. Significant cost differences between the intensive treatment arm and the routine care arm were observed only for glucose-lowering and lipid-lowering drugs.

The mean incremental annual costs of intensive treatment are presented in Table 3. Total incremental annual costs per patient over the 5-year time period averaged £92 (*β*_*t*otal-Cambridge_ = 92.0; SE_total-Cambridge_ = 115.4) using a GLM with a Gaussian distribution and an identity link. Incremental annual costs per patient for GP/nurse consultations and for metabolic/cardioprotective medication averaged £29 (*β*_consultation-Cambridge_ = 29.1; SE_consultation-Cambridge_ = 33.0) and £55 (*β*_medication-Cambridge_ = 54.6; SE_medication-Cambridge_ = 28.5), respectively. The incremental costs for other services were £6 (*β* = 5.7; SE = 89.1). About 4% of the variation in the total incremental costs was explained by the clustering of patients into surgeries (intraclass correlation coefficient = 0.036). A detailed analysis of the cost pattern over time showed that the cost difference varied considerably between the observation years (see Appendix 6 in Supplemental Materials found at doi:10.1016/j.jval.2017.05.018). Omitting the weighting factor or performing a complete case analysis (without imputed observation years) altered the results only marginally.

**Table 3:** Adjusted means of annual primary care costs according to IT and RC in the years 1–5*

	**Total**		**Consultations (ADDITION)**		**Medication (ADDITION)**		**Other primary care services**	
	**Mean**	**SE**	**Mean**	**SE**	**Mean**	**SE**	**Mean**	**SE**
IT	906.3	82.2	266.4	23.2	182.1	19.9	454.1	63.8
RC	814.3	81	237.2	23.5	127.6	20.4	448.3	62.3
Difference	92 (*β*_total_†)	115.4 (SE_total_†)	29.1 (*β*_consultation_†)	33 (SE_consultation_†)	54.6 (*β*_medication_†)	28.5 (SE_medication_†)	5.7	89.1

GP, general practitioner; HbA_1c_, glycated hemoglobin; IT, intensive treatment; RC, routine care; SE, standard error.

⁎ Generalized linear regression model with a Gaussian distribution and identitiy link with a main effect for the intervention and for time since diagnosis and an interaction term between intervention and time; adjusted for sex and age of diagnosis and baseline HbA_1c_; accounted for patients being clustered in GP surgeries and observations clustered in patients; model based on 841 observation years from 173 patients.

† Estimate used for long-term cost-effectiveness model.

### Long-Term Cost Effectiveness in ADDITION-UK

Table 4 presents the crude accumulated QALYs and costs over the 10-, 20-, and 30-year time horizon for ADDITION-UK and ADDITION-Cambridge. Table 5 presents the adjusted incremental QALYs, costs, and ICERs for ADDITION-UK and ADDITION-Cambridge. Because of larger cardiovascular risk factor reductions in Leicester [16], [35], incremental QALYs in ADDITION-UK were higher than in ADDITION-Cambridge. Nevertheless, because of higher implementation costs in Leicester incremental costs in ADDITION-UK were higher than in ADDITION-Cambridge. Resulting ICER point estimates for the 10-, 20-, and 30-year time horizon were £71,232/QALY, £28,444/QALY, and £27,549/QALY for ADDITION-UK and £96,570/QALY, £36,115/QALY, and £29,588/QALY, for ADDITION-Cambridge.

**Table 4:** Crude cumulative cost and QALYs according to IT and RC

**Time horizon**	**RC**					**IT**				
		**Crude costs**		**Crude QALYs**			**Crude costs**		**Crude QALYs**	
	**n**	**Mean**	**SE**	**Mean**	**SE**	**n**	**Mean**	**SE**	**Mean**	**SE**
*ADDITION-UK (Leicester and Cambridge)*										
10 y	501	6,157	332	6.45	0.08	498	7,256	879	6.40	0.09
20 y	501	11,175	867	9.32	0.21	498	12,392	1,614	9.16	0.23
30 y	501	13,181	1,325	10.08	0.30	498	14,308	2,110	9.82	0.31
*ADDITION-Cambridge*										
10 y	501	6,228	341	6.42	0.08	498	7,199	77,265	6.39	0.09
20 y	501	11,208	885	9.21	0.22	498	12,291	149,687	9.11	0.23
30 y	501	13,102	1,324	9.89	0.31	498	14,170	197,961	9.76	0.31

IT, intensive treatment; QALY, quality-adjusted life-year; RC, routine care; SE, standard error.

**Table 5:** Adjusted incremental costs and QALYs and ICER*

**Time horizon**	**Adjusted incremental cost (95% CI)**	**Adjusted incremental QALYs (95% CI)**	**ICER (£)**	***P***†
*ADDITION-UK (Leicester and Cambridge)*				
10 y	1,021 (920 to 1,120)	0.0143 (−0.0015 to 0.0294)	71,232	0.007
20 y	1,217 (1,029 to 1,406)	0.0428 (0.0034 to 0.0817)	28,444	0.535
30 y	1,311 (1,072 to 1,559)	0.0476 (0.0011 to 0.0932)	27,549	0.560
*ADDITION-Cambridge*				
10 y	927 (831 to 1,017)	0.0096 (−0.0079 to 0.0267)	96,570	0.009
20 y	1,086 (909 to 1,268)	0.0301 (−0.0144 to 0.0708)	36,115	0.395
30 y	1,157 (908 to 1,414)	0.0391 (−0.0107 to 0.0892)	29,588	0.500

CI, confidence interval; HbA_1c_, glycated hemoglobin; ICER, incremental cost-effectiveness ratio; QALY, quality-adjusted life-year; SE, standard error.

⁎ Means and SEs of QALYs and costs at patient level were used to conduct a bootstrap analysis (n = 500) adjusting for center, age at diagnosis, sex, and HbA_1c_ level at baseline.

† Probability that the ICER is <£30,000/QALY.

**Fig. 2:**
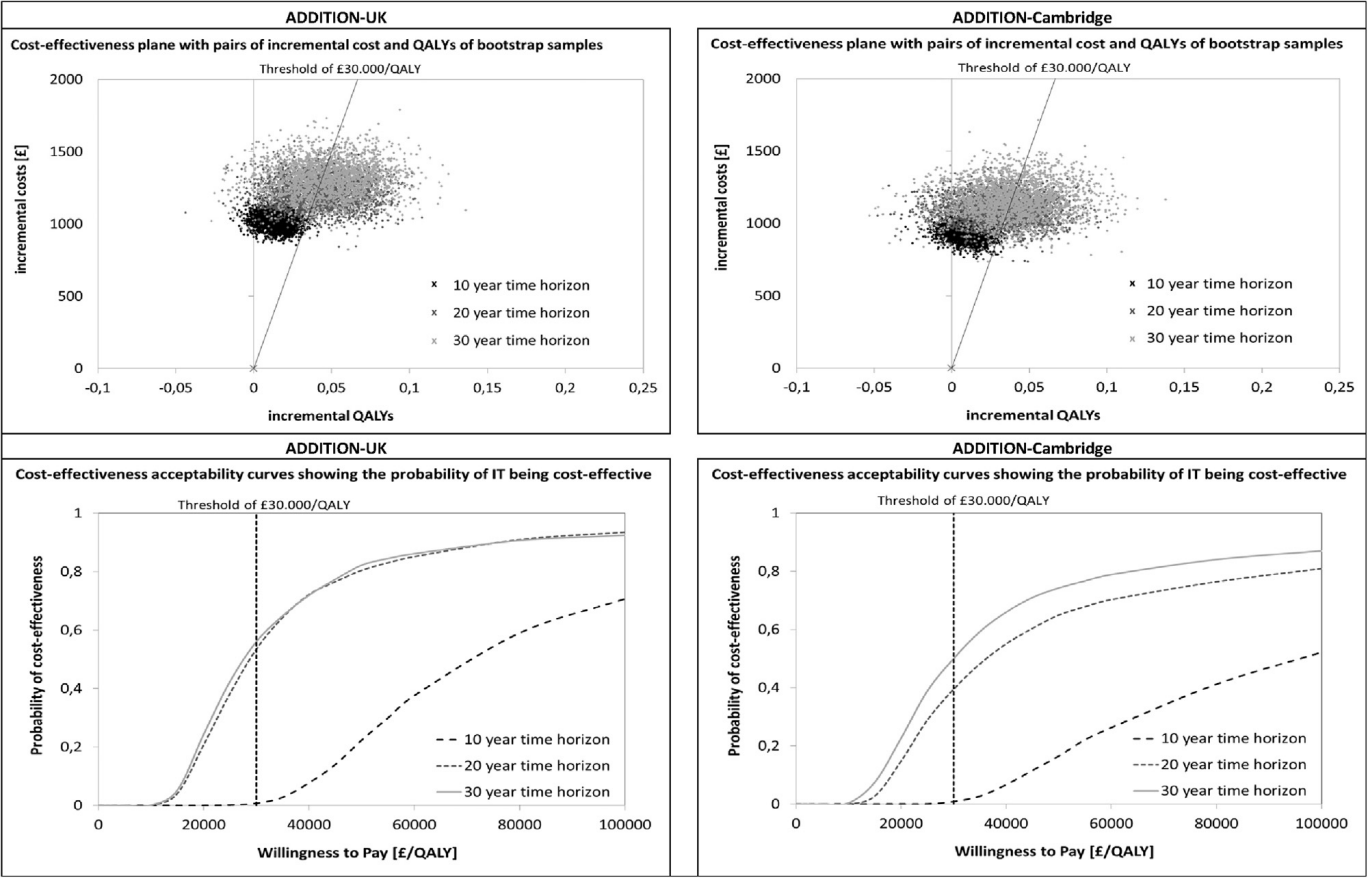
Cost-effectiveness planes showing pairs of 10-, 20-, and 30-year incremental costs and QALYs from bootstrap samples, and cost-effectiveness acceptability curves showing the probability of IT being more cost effective than RC on the basis of net benefit values from bootstrap samples over a time horizon of 10, 20, and 30 y. IT, intensive treatment; QALY, quality-adjusted life-year; RC, routine care.

Figure 2 shows the scatterplot of the 10-, 20-, and 30-year QALY and cost pairs of bootstrap replications in the cost-effectiveness plane and the cost-effectiveness acceptability curve. For all three time horizons, most of the points lie in the northeast quadrant. For ADDITION-UK 0.7%, 53.5%, and 56.0% and for ADDITION-Cambridge 0.9%, 39.5% and 50.0% are positioned below the £30,000/QALY WTP threshold.

### Sensitivity Analyses

**Fig. 3:**
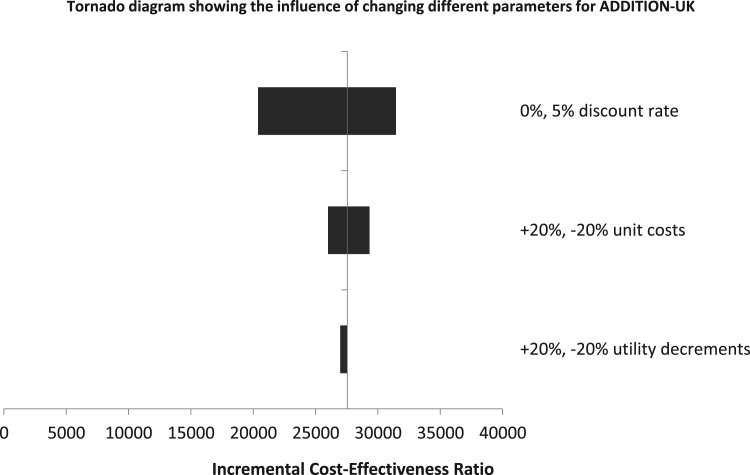
Tornado diagram showing the influence of changing different parameters that contribute to the ICER in long-term cost-effectiveness modeling analysis. Choice of discount rate has the greatest impact on the ICER (higher discount rate, unit costs, and lower utility decrements all associated with higher point estimate ICER). ICER, incremental cost-effectiveness ratio.

Incorporating total incremental primary care costs, including costs of consultations and other primary care services, yielded a 30-year ICER point estimate of £33,000/QALY for ADDITION-UK and of £38,000/QALY for ADDITION-Cambridge. The one-way sensitivity analysis with varying unit costs, discount rates, and utility decrement for ADDITON-UK is illustrated in the tornado diagram of Figure 3. It shows that for the specified ranges the ICER point estimate for ADDITION-UK lies close to or below the threshold of £30,000/QALY. Results from the sensitivity analysis for ADDITION-Cambridge are similar (not shown). Between 2010 and 2015/2016, relative prices increased by 44% for GP and nurse contacts and by 15% for unit costs for the treatment of diabetes complications, and they decreased by 41% for relevant cardiometabolic medications (see Appendix 4 in Supplemental Materials). The price change–adjusted models resulted in ICERs of £25,000/QALY for ADDITION-UK and £27,000/QALY for ADDITION-Cambridge.

## Discussion

There is uncertainty about the costs and the cost effectiveness of early intensive multifactorial treatment as delivered in the ADDITION trial. On the basis of electronic primary care records of a subsample of the trial cohort, we analyzed the incremental costs of delivered intensive treatment in ADDITION-Cambridge. Following an iterative framework of decision making in health care, we used these empirical cost estimates to update the previously published cost-effectiveness analysis for ADDITION-UK and present estimates for ADDITION-Cambridge. The results show that the intervention was delivered at lower costs than previously assumed and that there is a moderate likelihood that the intervention will be cost effective over a time horizon of 30 years.

The difficulty of decision making in the context of chronic diseases is that potential positive effects of treatment, that is, reduction in cardiovascular events and premature death, are likely to occur far from the time when interventions are delivered to patients. This issue is of particularly high relevance for interventions that target populations at a very early stage in disease progression, as in the case of treatment for individuals with type 2 diabetes detected by screening. Because decisions in health care often need to be made promptly and cannot be postponed until evidence from long-term trials is available, models that simulate the natural course of the disease, and with it the expected effects (QALYs) and costs, have been established as helpful tools [48]. Simulation models, however, rely on a set of assumptions and input parameters that crucially determine the results of the simulation.

To assess the cost effectiveness of the ADDITION intervention, we previously used the UKPDS outcomes model, which projects accumulated QALYs and costs over a 10-, 20-, and 30-year time horizon. This analysis showed that ICERs were only moderately sensitive to the used input parameters (unit costs for treatment of events, utility decrements for events, and discount rate), but highly sensitive to the assumptions on the costs of the intervention itself [17]. The input parameter for the incremental treatment costs was solely estimated on the trial protocol assuming 100% protocol adherence. To receive an empirical, trial-informed estimate, we therefore analyzed the electronic primary care records of a subset of the ADDITION-Cambridge trial cohort and used these data to update the long-term cost-effectiveness model.

The results of the empirical analysis show that the incremental per-patient costs for actually delivered consultations were lower than expected (£145 empirical vs. £311 protocol-based for years 1–5), but that the assumption for extra medication was appropriate (£54.6 empirical vs. £52.5 protocol- based annually; see Table 1). The former suggests that GPs did see their patients more often, but not to the extent for which they were reimbursed within the trial. The latter indicates that incremental costs for medication actually delivered were as high as the per-protocol estimated costs, which were based on the assumption of 100% protocol adherence with generic drug agents. This is surprising because we know that the protocol adherence was not perfect [18]. A possible explanation for this finding is that the reduction in costs resulting from the suboptimal medication adherence has been canceled out by an increase in costs resulting from the high usage of nongeneric drugs observed in both treatment arms. In more detailed analyses, for example, we observed that after the year 2003 when simvastatin went off patent, more than 35% of statin prescriptions were still for the much more expensive atorvastatin. Of note, costs for primary care services that were not directly related to the trial protocol were almost equal in both trial arms.

Revisiting the previously developed robust evaluation framework [17] with the empirical trial–informed cost estimates shows that the intervention has a moderate likelihood of being cost-effective over a time horizon of 30 years, assuming the higher UK NICE WTP threshold of £30,000/QALY. Our sensitivity analyses also indicated that the intervention might be cost effective with most recent prices.

This study also shows that empirical information on the incremental costs of the delivered intervention is invaluable for the economic evaluation of this trial. Unknown protocol adherence and the magnitude of generic drug usage can lead to a considerable over- or underestimation of incremental costs. Trialists should consider whether there could be value in measuring adherence to protocol when designing future pragmatic studies.

### Comparison with the Initial Cost-Effectiveness Analysis

The cost-effectiveness analysis in this study is based on a previously developed modeling framework [17]. A few minor methodological adaptations, however, have been made. Supported by the empirical data, annual costs for extra consultations in ADDITION-Cambridge were assumed to occur until year 5 and not only until year 3. Furthermore, the uncertainty of incremental costs was considered in the cost-effectiveness model by incorporating the SEs of the empirically derived incremental cost estimates in an additive manner. In the initial long-term cost-effectiveness analysis we also erroneously presumed that the mean costs for additional medication would be incurred until the end of the 10-, 20-, and 30-year simulation time horizon independently of individual simulated deaths of participants [17]. In this study, we took the more plausible assumption that costs for extra medication will occur until a person dies or reaches the end of the simulated time horizon. Applying this assumption to the previous cost-effectiveness analyses would have led to ICER point estimates of around £83,000/QALY, £32,000/QALY, and £30,000/QALY for a 10-, 20-, and 30-year simulation time horizon, respectively. The decrease in the ICER in our study can be explained by the lower frequency of extra consultations compared with the per-protocol assumed costs in the ADDITION-Cambridge sample (see Table 1).

### Strengths and Limitations

The main strength of this study is the use of empirical data from electronic primary care records from a subsample of the ADDITION-Cambridge trial sample. The use of these data provided a unique insight into the cost structure of intensive treatment as delivered in the ADDITION trial and allowed us to perform a detailed analysis of incremental cost components. This allowed us to revisit the cost-effectiveness analyses with the updated cost estimates using a previously developed robust evaluation framework and incorporated the uncertainty around the empirically derived cost estimates.

There are also some limitations that need to be taken into account. First and most importantly, the risk equations of the UKPDS outcomes model v1.3 were derived from a historical cohort followed from 1977 to 1997. Because the general quality of diabetes care has improved since then, the model overestimates the absolute cardiovascular disease risk in current populations. This finding was replicated in a previous validation study based on ADDITION data. Nevertheless, this validation study also showed that the model performed reasonably well in the prediction of incremental cardiovascular event rates in the ADDITION-UK sample [34]. Second, the input parameters for costs and utility decrements associated with the modeled events might be outdated und updating for inflation will not account for changes in relative prices. We therefore performed sensitivity analyses on these parameters, which showed that the results were only moderately sensitive toward variation in these parameters. Third, we had empirical information on primary care costs for only about 20% of the ADDITION-Cambridge trial cohort. We therefore kept the protocol-based assumptions for participants from Leicester in the analysis for ADDITION-UK, but performed separate analyses restricted to ADDITION-Cambridge participants. We further assigned mean cost estimates instead of individual-level costs to patients from Cambridge and Leicester. Fourth, because of the relatively small sample size, the clustering of patients into GP practices, and the nonavailability of information on resource utilization before randomization, the uncertainty around the cost estimates remained relatively large. Fifth, we had only empirical information on primary care contacts. We therefore used the risk factor profile of participants together with the UKPDS outcomes model to predict complications and costs (including hospital costs) associated with those complications. Nevertheless, we do not know whether the intervention provoked or prevented other unexpected care use that is not captured by the model and also not by our empirical primary care cost analyses. This shortcoming could have biased the cost estimates and ICERs in either direction. Sixth, we estimated incremental costs for medication on the basis of prescriptions issued. We, however, do not know how many of these were actually dispensed and we therefore probably overestimated the absolute (and incremental) costs for medications. Other limitations, such as the fact that the UKPDS outcomes model does not incorporate all diabetes-related complications and that the ADDITION-UK sample does not adequately represent UK ethnic diversity, limiting its external validity, have been discussed in detail by Tao et al. [17].

## Conclusions

Revisiting and correcting the initial cost-effectiveness analyses with empirical trial–informed cost estimates suggests that money spent on intensive treatment in individuals with type 2 diabetes detected by screening might be borderline cost effective according to conventional UK WTP thresholds. Nevertheless, the results need to be interpreted with caution because the projection of trial data over a long time horizon is almost always associated with substantial uncertainty.
