# Supplementary material for: Incremental Costs and Cost Effectiveness of Intensive Treatment in Individuals with Type 2 Diabetes Detected by Screening in the ADDITION-UK Trial: An Update with Empirical Trial–Based Cost Data
Source: Value Health. 2017 Dec;20(10):1288–98. doi: 10.1016/j.jval.2017.05.018 (PMC6086325; doi:10.1016/j.jval.2017.05.018)
Supplement: Supplementary file 1 — Supplementary material [file mmc1.pdf]

Appendix 1a: Baseline and five-year follow-up values for clinical variables for ADDITION-UK*

	Routine Care	Intensive treatment
	Adjusted difference* (SE) (follow up-baseline)	Adjusted difference* (SE) (follow up-baseline)
HbA_{1c} (%)	–0.25 (0.09)	–0.37 (0.09)
Total cholesterol (mmol/l)	–1.20 (0.07)	–1.30 (0.06)
Systolic blood pressure (mmHg)	–7.08 (1.13)	–7.32 (1.18)

* 85% of patients from are ADDITION UK belong to ADDITION Cambridge, compare Tao, L., et al. (2015). "Cost-effectiveness of intensive multifactorial treatment compared with routine care for individuals with screen-detected Type 2 diabetes: analysis of the ADDITION-UK cluster-randomized controlled trial." Diabet Med 6(10): 12711.

Appendix 1b: Hazard ratios for primary and secondary cardiovascular outcomes for ADDITION-UK*

	Routine Care	Intensive treatment
	HR (95%-CI)	
Myocardial infarction	ref	1.08 (0.40–2.94)
Stroke	ref	1.11 (0.52–2.35)
Revascularization	ref	0.68 (0.32–1.46)
CVD death	ref	0.45 (0.19–1.06)
All-cause death	ref	0.59 (0.35–0.98)
Composite cardiovascular events	ref	0.80 (0.55–1.17)

* 85% of patients from are ADDITION UK belong to ADDITION Cambridge, compare Griffin, S. J., et al. (2011). "Effect of early intensive multifactorial therapy on 5-year cardiovascular outcomes in individuals with type 2 diabetes detected by screening (ADDITION-Europe): a cluster-randomised trial." Lancet 378(9786): 156-167.

Appendix 1c: Odds ratios for microvascular outcomes for ADDITION-Leicester and ADDITION-Cambridge*

	Routine Care	Intensive treatment
	OR (95%-CI)	
ADDITION-Leicester		
Any albuminuria	ref	0.49 (0.21-1.15)
Any retinopathy	ref	0.90 (0.45-1.81)
Neuropathy	ref	1.76 (0.91-3.44)
ADDITION Cambridge		
Any albuminuria	ref	1.06 (0.74–1.53)
Any retinopathy	ref	0.77 (0.45–1.32)
Neuropathy	ref	0.55 (0.27–1.12)

* compare Sandbaek, A., et al. (2014). "Effect of early multifactorial therapy compared with routine care on microvascular outcomes at 5 years in people with screen-detected diabetes: a randomized controlled trial: the ADDITION-Europe Study." Diabetes Care 37(7): 2015-2023.
