# Supplementary material for: Incremental Costs and Cost Effectiveness of Intensive Treatment in Individuals with Type 2 Diabetes Detected by Screening in the ADDITION-UK Trial: An Update with Empirical Trial–Based Cost Data
Source: Value Health. 2017 Dec;20(10):1288–98. doi: 10.1016/j.jval.2017.05.018 (PMC6086325; doi:10.1016/j.jval.2017.05.018)
Supplement: Supplementary file 2 — Supplementary material [file mmc2.pdf]

Appendix 2: Unit cost (£, 2009/10 UK national level) and utility decrement for diabetes and diabetic complications modelled by the UKPDS outcomes model

	**Year of event**		**Subsequent years**			
	**Fatal**	**Non-fatal**		**Ref.**	**Utility decrement**	**Ref.**
Type 2 diabetes	-	494.5	494.5	[32]	-0.22	[31]
IHD	-	3558.4	1175.2	[32]	-0.09	[29]
MI	2295.6	6861.8	1129.8	[32]	-0.055	[29]
Heart failure	3968.4	3968.4	1391.1	[32]	-0.108	[29]
Stroke	5786.8	4196.9	793.4	[32]	-0.164	[29]
Re-vascular	-	4943.1	316.3	[30]	-0.059	[30]
Amputation	13664.2	13664.2	788.7	[32]	-0.28	[29]
Blindness	-	1791.7	758.9	[32]	-0.074	[29]
Renal failure	30599.2	30599.2	30599.2	[31]	-0.263	[29]
CVD death	3724.3	-	-	[30]	-	-

Costs extracted from the UKPDS study were based on participant hospital records and survey of 3488 UKPDS participants in 1996-97, from which inpatient and out-patient costs were predicted and updated to 2009/10 price year; compare Tao et al. [17]
