# Supplementary material for: Incremental Costs and Cost Effectiveness of Intensive Treatment in Individuals with Type 2 Diabetes Detected by Screening in the ADDITION-UK Trial: An Update with Empirical Trial–Based Cost Data
Source: Value Health. 2017 Dec;20(10):1288–98. doi: 10.1016/j.jval.2017.05.018 (PMC6086325; doi:10.1016/j.jval.2017.05.018)
Supplement: Supplementary file 3 — Supplementary material [file mmc3.pdf]

Appendix 3: Exploration of missing data

810 out of 31968 (32 variables x 999 observations) data were missing (2.5%). Missing data at follow-up was 10.9% (546 of 4995 data, 5 variables x 999 observations). Fifteen out of 32 baseline and follow-up variables had one or more observations missing. Missing data did not differ between treatment groups for 10 of the 15 variables. However, there was a statistically significant difference in the proportion of missing data between groups for baseline height (p=0.002), and follow-up measurements of total cholesterol (p=0.012), HDL (p<0.001), systolic blood pressure (p<0.001) and HbA1c (p=0.002). (Missingness in these follow-up parameters will be very highly correlated as they were intended to be collected at the same study visit).

Parameters associated with missing follow-up data on total and HDL cholesterol, SBP and HbA1c included gender, age, smoking status and HbA1c at diagnosis, ethnicity, although overall there is a mixed picture, with some likely spurious findings without any clinically plausible causation (e.g. a significant association between height and probability of HDL measurement being missing).

Based on the above, it may be reasonable to conclude that the data are not MCAR, but may be MAR. However, it is not (ever) possible to rule out MNAR. Given the low overall proportion of missing data (2.5% overall, 10.9% at follow-up), we conclude that MI is a reasonable approach to imputing missing data, allowing use of data that would otherwise be discarded in a complete case analysis.

STATA output log follows.

```
-----------------------------------------------------------------------------------------
      name:  <unnamed>
       log:  ExplorationofMissingness.smcl
  log type:  smcl
 opened on:  21 Feb 2017, 09:38:57

. import delimited "Unimputed_data.csv"
(33 vars, 999 obs)

.
. * change categorical to numeric
. gen male =0

. replace male = 1 if gender=="M"
(612 real changes made)

. drop gender

.
. gen diag_af = 0

. replace diag_af = 1 if diagnosisatrialfib == "Y"
(0 real changes made)

. drop diagnosisatrialfib

.
. gen diag_pvd = 0

. replace diag_pvd = 1 if diagnosispvd == "Y"
(0 real changes made)

. drop diagnosispvd

.
. misstable summ, gen(M_)
                                                               Obs<.
                                                +------------------------------
             |                                  | Unique
      Variable |     Obs=.     Obs>.     Obs<.  | values        Min         Max
```

```
  -------------+-------------------------------+------------------------------
        weight |         7               992  |    397       44.9        169.4
        height |       125               874  |    317      1.414         1.92
  diagnosisc~l |        19               980  |     65        2.2          9.2
  diagnosishdl |        23               976  |    154        .52          3.8
  diagnosiss~p |         3               996  |    254   90.33334          228
  diagnosis~1c |        21               978  |     90        4.1         15.3
   currentchol |        19               980  |     65        2.2          9.2
    currenthdl |        23               976  |    154        .52          3.8
  currentsysbp |         3               996  |    254   90.33334          228
  currenthba1c |        21               978  |     90        4.1         15.3
     fusmoking |        51               948  |      3          0            2
     futotchol |       109               890  |     53        2.1          8.8
         fuhdl |       138               861  |    152         .4          3.9
         fusbp |       124               875  |    214         93          220
       fuhba1c |       124               875  |     63        3.8         13.4
  ----------------------------------------------------------------------------

.
. * descriptive analysis of missing data
. * by trial group
. tabulate group M_weight, chi2

           |      (weight>=.)
     Group |         0          1 |     Total
-----------+----------------------+----------
         1 |       497          4 |       501
         2 |       495          3 |       498
-----------+----------------------+----------
     Total |       992          7 |       999

          Pearson chi2(1) =   0.1379   Pr = 0.710

. tabulate group M_height, chi2

           |      (height>=.)
     Group |         0          1 |     Total
-----------+----------------------+----------
         1 |       422         79 |       501
         2 |       452         46 |       498
-----------+----------------------+----------
     Total |       874        125 |       999

          Pearson chi2(1) =   9.7328   Pr = 0.002

. tabulate group M_diagnosischol, chi2

           |  (diagnosischol>=.)
     Group |         0          1 |     Total
-----------+----------------------+----------
         1 |       488         13 |       501
         2 |       492          6 |       498
-----------+----------------------+----------
     Total |       980         19 |       999

          Pearson chi2(1) =   2.5863   Pr = 0.108

. tabulate group M_diagnosishdl, chi2

           |   (diagnosishdl>=.)
     Group |         0          1 |     Total
-----------+----------------------+----------
         1 |       486         15 |       501
         2 |       490          8 |       498
-----------+----------------------+----------
     Total |       976         23 |       999

          Pearson chi2(1) =   2.1378   Pr = 0.144

. tabulate group M_diagnosissysbp, chi2

           |  (diagnosissysbp>=.)
     Group |         0          1 |     Total
-----------+----------------------+----------
         1 |       499          2 |       501
         2 |       497          1 |       498
-----------+----------------------+----------
```

```
     Total |       996          3 |       999 

          Pearson chi2(1) =   0.3283   Pr = 0.567

. tabulate group M_diagnosishba1c, chi2

           |  (diagnosishba1c>=.)
     Group |         0          1 |     Total
-----------+----------------------+----------
         1 |       489         12 |       501 
         2 |       489          9 |       498 
-----------+----------------------+----------
     Total |       978         21 |       999 

          Pearson chi2(1) =   0.4196   Pr = 0.517

. tabulate group M_currentchol, chi2

           |   (currentchol>=.)
     Group |         0          1 |     Total
-----------+----------------------+----------
         1 |       488         13 |       501 
         2 |       492          6 |       498 
-----------+----------------------+----------
     Total |       980         19 |       999 

          Pearson chi2(1) =   2.5863   Pr = 0.108

. tabulate group M_currenthdl, chi2

           |    (currenthdl>=.)
     Group |         0          1 |     Total
-----------+----------------------+----------
         1 |       486         15 |       501 
         2 |       490          8 |       498 
-----------+----------------------+----------
     Total |       976         23 |       999 

          Pearson chi2(1) =   2.1378   Pr = 0.144

. tabulate group M_currentsysbp, chi2

           |   (currentsysbp>=.)
     Group |         0          1 |     Total
-----------+----------------------+----------
         1 |       499          2 |       501 
         2 |       497          1 |       498 
-----------+----------------------+----------
     Total |       996          3 |       999 

          Pearson chi2(1) =   0.3283   Pr = 0.567

. tabulate group M_currenthba1c, chi2

           |   (currenthba1c>=.)
     Group |         0          1 |     Total
-----------+----------------------+----------
         1 |       489         12 |       501 
         2 |       489          9 |       498 
-----------+----------------------+----------
     Total |       978         21 |       999 

          Pearson chi2(1) =   0.4196   Pr = 0.517

. tabulate group M_fusmoking, chi2

           |    (fusmoking>=.)
     Group |         0          1 |     Total
-----------+----------------------+----------
         1 |       470         31 |       501 
         2 |       478         20 |       498 
-----------+----------------------+----------
     Total |       948         51 |       999 

          Pearson chi2(1) =   2.4311   Pr = 0.119

. tabulate group M_futotchol, chi2
```

```
           |    (futotchol>=.)
     Group |         0          1 |     Total
-----------+----------------------+----------
         1 |       434         67 |       501 
         2 |       456         42 |       498 
-----------+----------------------+----------
     Total |       890        109 |       999 

          Pearson chi2(1) =   6.2688   Pr = 0.012

. tabulate group M_fuhdl, chi2

           |      (fuhdl>=.)
     Group |         0          1 |     Total
-----------+----------------------+----------
         1 |       409         92 |       501 
         2 |       452         46 |       498 
-----------+----------------------+----------
     Total |       861        138 |       999 

          Pearson chi2(1) =  17.4720   Pr = 0.000

. tabulate group M_fusbp, chi2

           |      (fusbp>=.)
     Group |         0          1 |     Total
-----------+----------------------+----------
         1 |       417         84 |       501 
         2 |       458         40 |       498 
-----------+----------------------+----------
     Total |       875        124 |       999 

          Pearson chi2(1) =  17.5252   Pr = 0.000

. tabulate group M_fuhba1c, chi2

           |     (fuhba1c>=.)
     Group |         0          1 |     Total
-----------+----------------------+----------
         1 |       423         78 |       501 
         2 |       452         46 |       498 
-----------+----------------------+----------
     Total |       875        124 |       999 

          Pearson chi2(1) =   9.2103   Pr = 0.002

. 
. * association between missingness and baseline variables & observed outcomes
. logit M_fusmoking i.group i.ethnicity i.male age durationofdiabetes weight height i.dia
> g_af i.diag_pvd i.diagnosissmoking diagnosischol diagnosishdl diagnosissysbp diagnosish
> ba1c i.currentsmoking currentchol currenthdl currentsysbp currenthba1c preexistingihd p
> reexistingchf preexistingamp preexistingblind preexistingrenal preexistingstroke preexi
> stingmi

note: 1.ethnicity != 1 predicts failure perfectly
      1.ethnicity dropped and 75 obs not used

note: preexistingstroke != 0 predicts failure perfectly
      preexistingstroke dropped and 18 obs not used

note: preexistingmi != 0 predicts failure perfectly
      preexistingmi dropped and 43 obs not used

note: 2.ethnicity omitted because of collinearity
note: 3.ethnicity omitted because of collinearity
note: durationofdiabetes omitted because of collinearity
note: 0.diag_af omitted because of collinearity
note: 0.diag_pvd omitted because of collinearity
note: 1.currentsmoking omitted because of collinearity
note: 2.currentsmoking omitted because of collinearity
note: currentchol omitted because of collinearity
note: currenthdl omitted because of collinearity
note: currentsysbp omitted because of collinearity
note: currenthba1c omitted because of collinearity
note: preexistingihd omitted because of collinearity
note: preexistingchf omitted because of collinearity
```

```
note: preexistingamp omitted because of collinearity
note: preexistingblind omitted because of collinearity
note: preexistingrenal omitted because of collinearity
Iteration 0:   log likelihood = -104.67557
Iteration 1:   log likelihood = -100.22295
Iteration 2:   log likelihood = -99.016378
Iteration 3:   log likelihood = -99.012197
Iteration 4:   log likelihood = -99.012196

Logistic regression                               Number of obs   =        704
                                                  LR chi2(11)     =      11.33
                                                  Prob > chi2     =     0.4163
Log likelihood = -99.012196                       Pseudo R2       =     0.0541

------------------------------------------------------------------------------------
       M_fusmoking |      Coef.   Std. Err.      z    P>|z|     [95% Conf. Interval]
-------------------+----------------------------------------------------------------
           2.group |  -.4369343    .426696    -1.02   0.306    -1.273243    .3993744
                   |
         ethnicity |
                2  |          0  (empty)
                3  |          0  (empty)
                   |
            1.male |   .5959359   .6852547     0.87   0.384    -.7471386     1.93901
               age |   .0068199   .0327479     0.21   0.835    -.0573648    .0710047
durationofdiabetes |          0  (omitted)
            weight |   .0054153   .0146481     0.37   0.712    -.0232945    .0341252
            height |  -.6735042   3.406297    -0.20   0.843    -7.349724    6.002716
         0.diag_af |          0  (omitted)
        0.diag_pvd |          0  (omitted)
                   |
  diagnosissmoking |
                1  |  -.1189077   .5426201    -0.22   0.827    -1.182424    .9446082
                2  |   .9886518   .5306364     1.86   0.062    -.0513765     2.02868
                   |
     diagnosischol |  -.2643285   .1965389    -1.34   0.179    -.6495377    .1208807
      diagnosishdl |  -.4479791   .7774532    -0.58   0.564    -1.971759    1.075801
    diagnosissysbp |   .0037443   .0112236     0.33   0.739    -.0182535    .0257422
    diagnosishba1c |   .0145227   .1199981     0.12   0.904    -.2206692    .2497145
                   |
    currentsmoking |
                1  |          0  (omitted)
                2  |          0  (omitted)
                   |
       currentchol |          0  (omitted)
        currenthdl |          0  (omitted)
      currentsysbp |          0  (omitted)
      currenthba1c |          0  (omitted)
    preexistingihd |          0  (omitted)
    preexistingchf |          0  (omitted)
    preexistingamp |          0  (omitted)
  preexistingblind |          0  (omitted)
  preexistingrenal |          0  (omitted)
 preexistingstroke |          0  (omitted)
     preexistingmi |          0  (omitted)
             _cons |  -2.245222   6.202811    -0.36   0.717    -14.40251    9.912064
------------------------------------------------------------------------------------

. logit M_futotchol i.group i.ethnicity i.male age durationofdiabetes weight height i.dia
> g_af i.diag_pvd i.diagnosissmoking diagnosischol diagnosishdl diagnosissysbp diagnosish
> ba1c i.currentsmoking currentchol currenthdl currentsysbp currenthba1c preexistingihd p
> reexistingchf preexistingamp preexistingblind preexistingrenal preexistingstroke preexi
> stingmi

note: 2.ethnicity != 0 predicts failure perfectly
      2.ethnicity dropped and 11 obs not used

note: durationofdiabetes omitted because of collinearity
note: 0.diag_af omitted because of collinearity
note: 0.diag_pvd omitted because of collinearity
note: 1.currentsmoking omitted because of collinearity
note: 2.currentsmoking omitted because of collinearity
note: currentchol omitted because of collinearity
note: currenthdl omitted because of collinearity
note: currentsysbp omitted because of collinearity
note: currenthba1c omitted because of collinearity
note: preexistingihd omitted because of collinearity
```

```
note: preexistingchf omitted because of collinearity
note: preexistingamp omitted because of collinearity
note: preexistingblind omitted because of collinearity
note: preexistingrenal omitted because of collinearity
Iteration 0:   log likelihood = -86.740425
Iteration 1:   log likelihood = -78.864431
Iteration 2:   log likelihood = -74.632087
Iteration 3:   log likelihood = -74.577762
Iteration 4:   log likelihood = -74.577679
Iteration 5:   log likelihood = -74.577679

Logistic regression                               Number of obs   =        829
                                                  LR chi2(14)     =      24.33
                                                  Prob > chi2     =     0.0418
Log likelihood = -74.577679                       Pseudo R2       =     0.1402

------------------------------------------------------------------------------------
       M_futotchol |      Coef.   Std. Err.      z    P>|z|     [95% Conf. Interval]
-------------------+----------------------------------------------------------------
           2.group |   .6055944   .5283212     1.15   0.252    -.4298962    1.641085
                   |
         ethnicity |
                2  |          0  (empty)
                3  |   1.855084   1.214619     1.53   0.127    -.5255253    4.235694
                   |
            1.male |  -1.774157   .7614911    -2.33   0.020    -3.266652   -.2816621
               age |   .1278512   .0527805     2.42   0.015     .0244033    .2312991
durationofdiabetes |          0  (omitted)
            weight |   .0145182   .0170165     0.85   0.394    -.0188336      .04787
            height |   4.867089   3.936642     1.24   0.216    -2.848587    12.58277
         0.diag_af |          0  (omitted)
        0.diag_pvd |          0  (omitted)
                   |
  diagnosissmoking |
                1  |   1.023356    .681976     1.50   0.133    -.3132925    2.360004
                2  |   1.884861   .7276166     2.59   0.010     .4587584    3.310963
                   |
     diagnosischol |   .1628792   .2125308     0.77   0.443    -.2536736     .579432
      diagnosishdl |   .4883364   .6982475     0.70   0.484    -.8802036    1.856876
    diagnosissysbp |   .0143141    .011474     1.25   0.212    -.0081745    .0368027
    diagnosishba1c |   .1743563   .1285212     1.36   0.175    -.0775407    .4262532
                   |
    currentsmoking |
                1  |          0  (omitted)
                2  |          0  (omitted)
                   |
       currentchol |          0  (omitted)
        currenthdl |          0  (omitted)
      currentsysbp |          0  (omitted)
      currenthba1c |          0  (omitted)
    preexistingihd |          0  (omitted)
    preexistingchf |          0  (omitted)
    preexistingamp |          0  (omitted)
  preexistingblind |          0  (omitted)
  preexistingrenal |          0  (omitted)
 preexistingstroke |   .0475647   .1322037     0.36   0.719    -.2115498    .3066792
     preexistingmi |   .0004308   .0812079     0.01   0.996    -.1587337    .1595952
             _cons |  -26.72693   8.207629    -3.26   0.001    -42.81359   -10.64027
------------------------------------------------------------------------------------

. logit M_fuhdl i.group i.ethnicity i.male age durationofdiabetes weight height i.diag_af
>  i.diag_pvd i.diagnosissmoking diagnosischol diagnosishdl diagnosissysbp diagnosishba1c
>  i.currentsmoking currentchol currenthdl currentsysbp currenthba1c preexistingihd preex
> istingchf preexistingamp preexistingblind preexistingrenal preexistingstroke preexistin
> gmi

note: 2.ethnicity != 0 predicts failure perfectly
      2.ethnicity dropped and 11 obs not used

note: durationofdiabetes omitted because of collinearity
note: 0.diag_af omitted because of collinearity
note: 0.diag_pvd omitted because of collinearity
note: 1.currentsmoking omitted because of collinearity
note: 2.currentsmoking omitted because of collinearity
note: currentchol omitted because of collinearity
note: currenthdl omitted because of collinearity
note: currentsysbp omitted because of collinearity
```

```
note: currenthba1c omitted because of collinearity
note: preexistingihd omitted because of collinearity
note: preexistingchf omitted because of collinearity
note: preexistingamp omitted because of collinearity
note: preexistingblind omitted because of collinearity
note: preexistingrenal omitted because of collinearity
Iteration 0:   log likelihood = -145.02106
Iteration 1:   log likelihood = -137.00905
Iteration 2:   log likelihood =  -135.5593
Iteration 3:   log likelihood = -135.55551
Iteration 4:   log likelihood =  -135.5555

Logistic regression                               Number of obs   =        829
                                                  LR chi2(14)     =      18.93
                                                  Prob > chi2     =     0.1676
Log likelihood =  -135.5555                       Pseudo R2       =     0.0653

------------------------------------------------------------------------------------
           M_fuhdl |      Coef.   Std. Err.      z    P>|z|     [95% Conf. Interval]
-------------------+----------------------------------------------------------------
           2.group |  -.5516725   .3616837    -1.53   0.127     -1.26056    .1572144
                   |
         ethnicity |
                2  |          0  (empty)
                3  |  -.3972339   1.093944    -0.36   0.717    -2.541325    1.746857
                   |
            1.male |  -1.301736   .5508324    -2.36   0.018    -2.381348   -.2221248
               age |   .0173194   .0294563     0.59   0.557    -.0404139    .0750528
durationofdiabetes |          0  (omitted)
            weight |   .0046728   .0120955     0.39   0.699     -.019034    .0283795
            height |   5.619519   2.861427     1.96   0.050     .0112243    11.22781
         0.diag_af |          0  (omitted)
        0.diag_pvd |          0  (omitted)
                   |
  diagnosissmoking |
                1  |   .5523239   .4510155     1.22   0.221    -.3316503    1.436298
                2  |   1.140725   .4933152     2.31   0.021     .1738448    2.107605
                   |
     diagnosischol |    -.16806    .160809    -1.05   0.296    -.4832399    .1471199
      diagnosishdl |   .5487335   .5323884     1.03   0.303    -.4947285    1.592196
    diagnosissysbp |   .0105278   .0088739     1.19   0.235    -.0068648    .0279203
    diagnosishba1c |   .0532259   .1014626     0.52   0.600    -.1456373     .252089
                   |
    currentsmoking |
                1  |          0  (omitted)
                2  |          0  (omitted)
                   |
       currentchol |          0  (omitted)
        currenthdl |          0  (omitted)
      currentsysbp |          0  (omitted)
      currenthba1c |          0  (omitted)
    preexistingihd |          0  (omitted)
    preexistingchf |          0  (omitted)
    preexistingamp |          0  (omitted)
  preexistingblind |          0  (omitted)
  preexistingrenal |          0  (omitted)
 preexistingstroke |  -.0110728   .1450093    -0.08   0.939    -.2952859    .2731402
     preexistingmi |  -.0362379   .0844939    -0.43   0.668    -.2018428    .1293671
             _cons |  -15.20218   5.366486    -2.83   0.005     -25.7203    -4.68406
------------------------------------------------------------------------------------

. logit M_fusbp i.group i.ethnicity i.male age durationofdiabetes weight height i.diag_af
>  i.diag_pvd i.diagnosissmoking diagnosischol diagnosishdl diagnosissysbp diagnosishba1c
>  i.currentsmoking currentchol currenthdl currentsysbp currenthba1c preexistingihd preex
> istingchf preexistingamp preexistingblind preexistingrenal preexistingstroke preexistin
> gmi

note: 2.ethnicity != 0 predicts failure perfectly
      2.ethnicity dropped and 11 obs not used

note: preexistingstroke != 0 predicts failure perfectly
      preexistingstroke dropped and 18 obs not used

note: preexistingmi != 0 predicts failure perfectly
      preexistingmi dropped and 44 obs not used

note: durationofdiabetes omitted because of collinearity
```

```
note: 0.diag_af omitted because of collinearity
note: 0.diag_pvd omitted because of collinearity
note: 1.currentsmoking omitted because of collinearity
note: 2.currentsmoking omitted because of collinearity
note: currentchol omitted because of collinearity
note: currenthdl omitted because of collinearity
note: currentsysbp omitted because of collinearity
note: currenthba1c omitted because of collinearity
note: preexistingihd omitted because of collinearity
note: preexistingchf omitted because of collinearity
note: preexistingamp omitted because of collinearity
note: preexistingblind omitted because of collinearity
note: preexistingrenal omitted because of collinearity
Iteration 0:   log likelihood = -81.567845
Iteration 1:   log likelihood = -68.508296
Iteration 2:   log likelihood = -60.419285
Iteration 3:   log likelihood = -60.035641
Iteration 4:   log likelihood = -60.032374
Iteration 5:   log likelihood = -60.032374

Logistic regression                               Number of obs   =        767
                                                  LR chi2(12)     =      43.07
                                                  Prob > chi2     =     0.0000
Log likelihood = -60.032374                       Pseudo R2       =     0.2640

------------------------------------------------------------------------------------
           M_fusbp |      Coef.   Std. Err.      z    P>|z|     [95% Conf. Interval]
-------------------+----------------------------------------------------------------
           2.group |  -.9131446    .605406    -1.51   0.131    -2.099719    .2734295
                   |
         ethnicity |
                2  |          0  (empty)
                3  |   4.315916   .9033088     4.78   0.000     2.545463    6.086368
                   |
            1.male |  -.8184545   .7601726    -1.08   0.282    -2.308365    .6714565
               age |   .0980135   .0456757     2.15   0.032     .0084907    .1875362
durationofdiabetes |          0  (omitted)
            weight |  -.0030261    .017827    -0.17   0.865    -.0379664    .0319142
            height |   1.316118   4.518181     0.29   0.771    -7.539354    10.17159
         0.diag_af |          0  (omitted)
        0.diag_pvd |          0  (omitted)
                   |
  diagnosissmoking |
                1  |    .352746   .8390663     0.42   0.674    -1.291794    1.997286
                2  |   1.800109   .7406252     2.43   0.015       .34851    3.251707
                   |
     diagnosischol |    .143628   .2561239     0.56   0.575    -.3583656    .6456216
      diagnosishdl |   1.000652    .861717     1.16   0.246    -.6882825    2.689586
    diagnosissysbp |   -.010975   .0163859    -0.67   0.503    -.0430907    .0211407
    diagnosishba1c |   .3939303   .1562118     2.52   0.012     .0877608    .7000999
                   |
    currentsmoking |
                1  |          0  (omitted)
                2  |          0  (omitted)
                   |
       currentchol |          0  (omitted)
        currenthdl |          0  (omitted)
      currentsysbp |          0  (omitted)
      currenthba1c |          0  (omitted)
    preexistingihd |          0  (omitted)
    preexistingchf |          0  (omitted)
    preexistingamp |          0  (omitted)
  preexistingblind |          0  (omitted)
  preexistingrenal |          0  (omitted)
 preexistingstroke |          0  (omitted)
     preexistingmi |          0  (omitted)
             _cons |  -16.07387   8.862739    -1.81   0.070    -33.44452    1.296782
------------------------------------------------------------------------------------

. logit M_fuhba1c i.group i.ethnicity i.male age durationofdiabetes weight height i.diag_
> af i.diag_pvd i.diagnosissmoking diagnosischol diagnosishdl diagnosissysbp diagnosishba
> 1c i.currentsmoking currentchol currenthdl currentsysbp currenthba1c preexistingihd pre
> existingchf preexistingamp preexistingblind preexistingrenal preexistingstroke preexist
> ingmi

note: 2.ethnicity != 0 predicts failure perfectly
      2.ethnicity dropped and 11 obs not used
```

```
note: preexistingmi != 0 predicts failure perfectly
      preexistingmi dropped and 47 obs not used

note: durationofdiabetes omitted because of collinearity
note: 0.diag_af omitted because of collinearity
note: 0.diag_pvd omitted because of collinearity
note: 1.currentsmoking omitted because of collinearity
note: 2.currentsmoking omitted because of collinearity
note: currentchol omitted because of collinearity
note: currenthdl omitted because of collinearity
note: currentsysbp omitted because of collinearity
note: currenthba1c omitted because of collinearity
note: preexistingihd omitted because of collinearity
note: preexistingchf omitted because of collinearity
note: preexistingamp omitted because of collinearity
note: preexistingblind omitted because of collinearity
note: preexistingrenal omitted because of collinearity
Iteration 0:   log likelihood = -136.75016
Iteration 1:   log likelihood = -133.02949
Iteration 2:   log likelihood = -118.99277
Iteration 3:   log likelihood = -118.70479
Iteration 4:   log likelihood = -118.70386
Iteration 5:   log likelihood = -118.70386

Logistic regression                               Number of obs   =        782
                                                  LR chi2(13)     =      36.09
                                                  Prob > chi2     =     0.0006
Log likelihood = -118.70386                       Pseudo R2       =     0.1320

------------------------------------------------------------------------------------
         M_fuhba1c |      Coef.   Std. Err.      z    P>|z|     [95% Conf. Interval]
-------------------+----------------------------------------------------------------
           2.group |  -.0584053   .3870503    -0.15   0.880    -.8170098    .7001993
                   |
         ethnicity |
                2  |          0  (empty)
                3  |   3.242644   .6263551     5.18   0.000     2.015011    4.470277
                   |
            1.male |  -1.271986   .5590786    -2.28   0.023     -2.36776   -.1762125
               age |   .0834566   .0327163     2.55   0.011     .0193338    .1475794
durationofdiabetes |          0  (omitted)
            weight |   .0091622   .0123752     0.74   0.459    -.0150929    .0334172
            height |    4.75904   3.074451     1.55   0.122    -1.266772    10.78485
         0.diag_af |          0  (omitted)
        0.diag_pvd |          0  (omitted)
                   |
  diagnosissmoking |
                1  |   .4008047   .5096235     0.79   0.432    -.5980389    1.399648
                2  |   1.386312   .5194204     2.67   0.008     .3682664    2.404357
                   |
     diagnosischol |   .0333093   .1715088     0.19   0.846    -.3028419    .3694604
      diagnosishdl |   .5690557   .5785333     0.98   0.325    -.5648486     1.70296
    diagnosissysbp |   .0113376   .0096713     1.17   0.241    -.0076177     .030293
    diagnosishba1c |   .1466052   .1065584     1.38   0.169    -.0622454    .3554557
                   |
    currentsmoking |
                1  |          0  (omitted)
                2  |          0  (omitted)
                   |
       currentchol |          0  (omitted)
        currenthdl |          0  (omitted)
      currentsysbp |          0  (omitted)
      currenthba1c |          0  (omitted)
    preexistingihd |          0  (omitted)
    preexistingchf |          0  (omitted)
    preexistingamp |          0  (omitted)
  preexistingblind |          0  (omitted)
  preexistingrenal |          0  (omitted)
 preexistingstroke |   .0277705   .1338047     0.21   0.836    -.2344818    .2900228
     preexistingmi |          0  (omitted)
             _cons |  -20.91242   6.064717    -3.45   0.001    -32.79905   -9.025794
------------------------------------------------------------------------------------

.
. log close
      name:  <unnamed>
```

```
      log:  ExplorationofMissingness.smcl
 log type:  smcl
closed on:  21 Feb 2017, 09:38:58
```
