# Supplementary material for: Incremental Costs and Cost Effectiveness of Intensive Treatment in Individuals with Type 2 Diabetes Detected by Screening in the ADDITION-UK Trial: An Update with Empirical Trial–Based Cost Data
Source: Value Health. 2017 Dec;20(10):1288–98. doi: 10.1016/j.jval.2017.05.018 (PMC6086325; doi:10.1016/j.jval.2017.05.018)
Supplement: Supplementary file 4 — Supplementary material [file mmc4.pdf]

Appendix 6: Relative price changes for chosen relevant services from 2009/2010 to 2015/2016

					Year 2009/2010				Year 2015/2016					relative price change	
		relative frequency*	cost weight#	combined weight ⌐	2009/10	NHS currency code	Source	Location	2015/16	NHS currency code	Source	Location	Notes	relative price change	weighted price change
	**Source: the National Schedule of Reference Costs for NHS Trusts (NSRC) for 2009/2010 and 2015/2016**														
Hospitalisations for complications	Hospitalisation for MI	0.16	0.05	0.03	£1,415.00	EB10Z	https://www.gov.uk/government/uploads/system/uploads/attachment_data/file/216308/dh_123455.xls	sheet 'Total - HRGs' cell D315	£1,497.00	weighted av of EB10A, EB10B, EB10C, EB10D, EB10E	https://www.gov.uk/government/uploads/system/uploads/attachment_data/file/577084/National_schedule_of_reference_costs_-_main_schedule.xlsx	sheet 'Total HRG's', weighted average of C571:D575		0.06	
	Hospitalisation for Stroke	0.36	0.09	0.14	£2,448.00	AA22Z	https://www.gov.uk/government/uploads/system/uploads/attachment_data/file/216308/dh_123455.xls	sheet 'Total - HRGs' cell D29	£3,233.00	weighted av of AA22C,D,E,F,G and AA35A,B,C,D,E,F	https://www.gov.uk/government/uploads/system/uploads/attachment_data/file/216308/dh_123455.xls	sheet 'Total HRG's', weighted average of C7:D11, C53:D58	Not directly comparable - in 2009/10, no specific 'stroke' HRG ('non-transient stroke or CVA, nervous system infections or encephalopathy')	0.32	
	Hospitalisation for Revascularization	0.24	0.44	0.48	£11,344.79	EA51Z	https://www.gov.uk/government/uploads/system/uploads/attachment_data/file/216308/dh_123455.xls	sheet 'Total - HRGs' cell D300	£14,326.68	weighted av of ED22A,B,C and ED23A,B,C	https://www.gov.uk/government/uploads/system/uploads/attachment_data/file/216308/dh_123455.xls	sheet 'Total HRG's', weighted average of C631:D636	Not directly comparable - in 2009/10, no specific 'Coronary Artery Bypass Graft with valve replacement or repair')	0.26	
	Hospitalisation for Heart Failure	0.18	0.17	0.13	£2,405.64	EB03H	https://www.gov.uk/government/uploads/system/uploads/attachment_data/file/216308/dh_123455.xls	sheet 'Total - HRGs' cell D304	£2,066.10	weighted av of EB03A,B,C,D,E	https://www.gov.uk/government/uploads/system/uploads/attachment_data/file/216308/dh_123455.xls	sheet 'Total HRG's', weighted average of C546:D550	Not directly comparable - in 2009/10, no specific 'Heart Failure or Shock with CC')	-0.14	
	Hospitalisation for Amputation	0.03	0.83	0.11	£10,092.77	weighted av of QZ11A,B	https://www.gov.uk/government/uploads/system/uploads/attachment_data/file/216308/dh_123455.xls	sheet 'Total - HRGs', weighted average of C1031:D1032	£8,748.84	weighted av of YQ20A:YQ26C	https://www.gov.uk/government/uploads/system/uploads/attachment_data/file/216308/dh_123455.xls	sheet 'Total HRG's', weighted average of C2696:D2710	Not directly comparable	-0.13	
	Hospitalisation for Renal Failure	0.02	0.97	0.10	£1,997.76	weighted av of LA08A,B,C,E,F	https://www.gov.uk/government/uploads/system/uploads/attachment_data/file/216308/dh_123455.xls	sheet 'Total - HRGs', weighted average of C820:D824	£2,147.16	weighted av of LA08G:LA08P	https://www.gov.uk/government/uploads/system/uploads/attachment_data/file/216308/dh_123455.xls	sheet 'Total HRG's', weighted average of C1511:D1518	Not directly comparable	0.07	
														**Average**	**0.15**
	**Source:PSSRU Unit Costs of Health and Social Care 2010 and 2016**														
Consultations with GPs and nurses	General Practitioner (per 17 min clinic visit)	0.51	0.63	0.64	£44.00	-	http://www.pssru.ac.uk/project-pages/unit-costs/2010/	Page 167	£65.00	-	http://www.pssru.ac.uk/project-pages/unit-costs/2015/	Page 177	-	0.48	
	Nurse contact (per hour)	0.49	0.37	0.36	£26.00	-	http://www.pssru.ac.uk/	Page 164	£36.00	-	http://www.pssru.ac.uk/	Page 174	-	0.38	
														**Average**	**0.44**
	**British National Formulary (BNF) for 2009/2010 and 2015/2016**														
Medications (glucose lowering, lipid lowering & blood pressure lowering)	Gliclazide	0.02	0.01	0.01	£1.45	-	BNF59 March 2010	80mg 60 tab pack non-prop, P413	£4.38	-	BNF71 March 2016	80mg 60 tab pack diamicron, P622	-	2.02	
	Glimepiride	0.01	0.02	0.00	£2.74	-	BNF59 March 2010	4mg 30 tab pack non-prop, P414	£1.41	-	BNF71 March 2016	4mg 30 tab pack non-prop, P623	-	-0.49	
	Metformin	0.17	0.01	0.05	£1.33	-	BNF59 March 2010	850mg 56 tab pack non-prop, p415	£1.94	-	BNF71 March 2016	850mg 56 tab pack non-prop, p604	-	0.46	
	Nateglinide	0.00	0.14	0.01	£25.88	-	BNF59 March 2010	180mg 84 tab pack Starlix, p418	£29.76	-	BNF71 March 2016	180mg 84 tab pack Starlix, p618	-	0.15	
	Pioglitazone	0.00	0.20	0.01	£36.96	-	BNF59 March 2010	45mg 28 tab pack, Actos,P418	£1.60	-	BNF71 March 2016	45mg 28 tab pack, non-prop p624	-	-0.96	
	Rosiglitazone	0.02	0.16	0.10	£30.00	-	BNF59 March 2010	8mg 28 tab pack, Avandia, P419	£35.19	-	withdrawn from EU market 2010. Assumed 2010 price inflated to 2015 using HCHS prices index (=17.3% increase)		-	0.17	
		0.00	0.00												
	Atorvastatin	0.07	0.15	0.42	£28.21	-	BNF59 March 2010	80mg 28 tab pack, Lipitor p156	£2.79	-	BNF71 March 2016	80mg 28 tab pack, non-prop, P180	-	-0.90	
	Fluvastatin	0.01	0.05	0.03	£9.97	-	BNF59 March 2010	40mg 28 cap pack, non-	£7.42	-	BNF71 March 2016	40mg 28 cap pack, non-	-	-0.26	
	Pravastatin	0.01	0.02	0.01	£3.02	-	BNF59 March 2010	40mg 28 tab pack non-prop, p157	£1.80	-	BNF71 March 2016	40mg 28 tab pack non-prop, p181	-	-0.40	
	Rosuvastatin	0.02	0.16	0.12	£29.69	-	BNF59 March 2010	40mg 28 tab pack Crestor, p157	£29.69	-	BNF71 March 2016	40mg 28 tab pack Crestor, p182	-	0.00	
	Simvastatin	0.20	0.02	0.14	£3.27	-	BNF59 March 2010	80mg 28 tab pack, non-prop p157	£1.99	-	BNF71 March 2016	80mg 28 tab pack, non-prop p182	-	-0.39	
		0.00	0.00												
	Amlodipine	0.06	0.01	0.02	£1.26	-	BNF59 March 2010	10mg 28 tab pack, non-prop p124	£0.98	-	BNF71 March 2016	10mg 28 tab pack, non-prop p149	-	-0.22	
	Atenolol	0.08	0.01	0.02	£0.92	-	BNF59 March 2010	100mg 28 tab pack, non-prop, p96	£0.92	-	BNF71 March 2016	100mg 28 tab pack, non-prop, p146	-	0.00	
	Bendroflumethiazide	0.07	0.01	0.01	£0.94	-	BNF59 March 2010	5mg 28 non-prop, p82	£1.40	-	BNF71 March 2016	5mg 28 non-prop, p160	-	0.49	
	Lisinopril	0.10	0.01	0.02	£1.27	-	BNF59 March 2010	20mg 28 tab pack, non-prop, p114	£1.05	-	BNF71 March 2016	20mg 28 tab pack, non-prop, p129	-	-0.17	
	Ramipril	0.07	0.01	0.02	£1.46	-	BNF59 March 2010	10mg 28 cap pack, non-prop p116	£1.33	-	BNF71 March 2016	10mg 28 cap pack, non-prop p132	-	-0.09	
	Furosemide	0.04	0.00	0.01	£0.90	-	BNF59 March 2010	40mg 28, non-prop, p83	£0.85	-	BNF71 March 2016	40mg 28, non-prop, p202	-	-0.06	
	Perindopril	0.03	0.02	0.02	£2.74	-	BNF59 March 2010	erbumine. 8mg 30 tab pack non-prop, p115	£1.39	-	BNF71 March 2016	erbumine. 8mg 30 tab pack non-prop, p131	-	-0.49	
														**Average**	**-0.41**

* observed relative frequency in the study, # relative cost weight, ⌐ combination of relative observed frequency and relative cost weight
