# Supplementary material for: Incremental Costs and Cost Effectiveness of Intensive Treatment in Individuals with Type 2 Diabetes Detected by Screening in the ADDITION-UK Trial: An Update with Empirical Trial–Based Cost Data
Source: Value Health. 2017 Dec;20(10):1288–98. doi: 10.1016/j.jval.2017.05.018 (PMC6086325; doi:10.1016/j.jval.2017.05.018)
Supplement: Supplementary file 5 — Supplementary material [file mmc5.pdf]

Appendix 5: Resource utilization according to intensive treatment (IT) and routine care (RC)

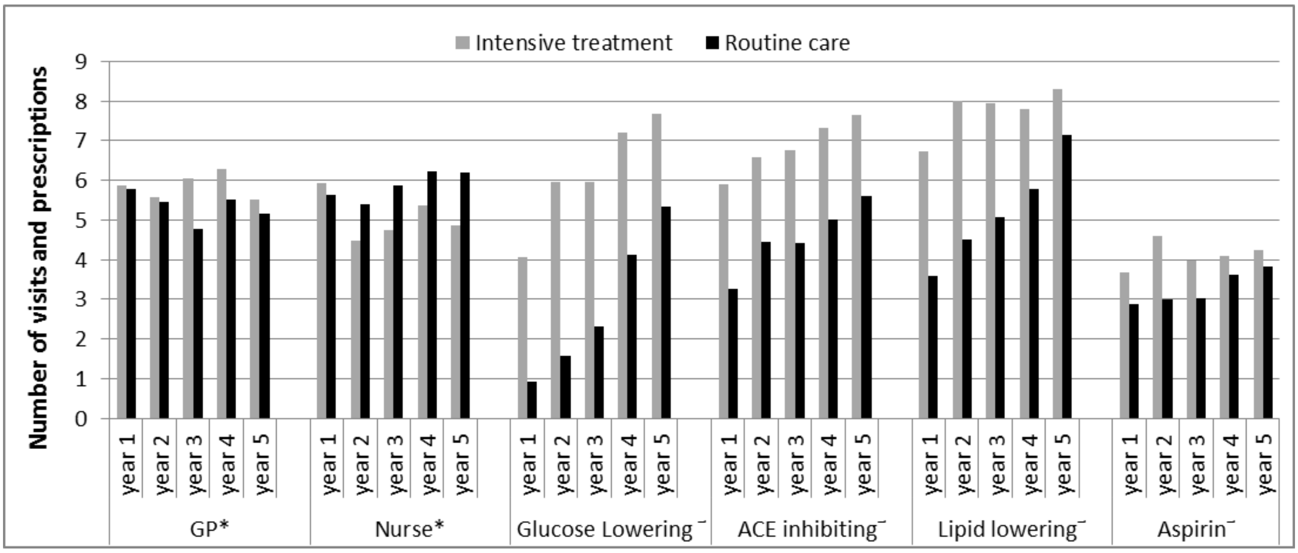

* number of annual contacts; ¬ number of annual prescriptions
