# Supplementary material for: Incremental Costs and Cost Effectiveness of Intensive Treatment in Individuals with Type 2 Diabetes Detected by Screening in the ADDITION-UK Trial: An Update with Empirical Trial–Based Cost Data
Source: Value Health. 2017 Dec;20(10):1288–98. doi: 10.1016/j.jval.2017.05.018 (PMC6086325; doi:10.1016/j.jval.2017.05.018)
Supplement: Supplementary file 6 — Supplementary material [file mmc6.pdf]

Appendix 6: Adjusted means of annual primary costs according to Intensive Treatment and Routine Care in the years 1-5 #

		year 1		year 2		year 3		year 4		year 5		overall	
		**mean**	**(SE)**	**mean**	**(SE)**	**mean**	**(SE)**	**mean**	**(SE)**	**mean**	**(SE)**	**mean**	**(SE)**
Total *	IT	809.9	(92.1)	836.9	(92)	886.7	(91.9)	992.8	(92)	1005.1	(92.6)	906.3	(82.2)
	RC	688.0	(90.2)	706.9	(90.4)	847.0	(90.3)	871.2	(90.8)	958.3	(91.1)	814.3	(81)
	dif.	**122.0**	**(128.6)**	**130.0**	**(128.7)**	**39.7**	**(128.9)**	**121.5**	**(129.3)**	**46.8**	**(129.9)**	**92.0**[a]	**(115.4)**[b]
Total **	IT	794.2	(81.1)	822.7	(83.9)	867.2	(88.4)	972.8	(99.2)	1001.0	(102.8)	887.9	(80.7)
	RC	693.6	(69.3)	703.8	(70.5)	846.7	(84.7)	869.4	(87.5)	952.1	(96.1)	806.9	(72.3)
	dif.	**100.6**	**-**	**118.9**	**-**	**20.5**	**-**	**103.4**	**-**	**48.9**	**-**	**80.9**	**-**
Consultations (ADDITION) *	IT	273.1	(28.6)	241.3	(28.6)	275.1	(28.5)	285.0	(28.6)	257.3	(28.9)	266.4	(23.2)
	RC	241.5	(28.4)	232.4	(28.5)	216.6	(28.4)	231.3	(28.7)	264.5	(28.8)	237.2	(23.5)
	dif.	31.6	(40.1)	8.9	(40.2)	58.6	(40.3)	53.7	(40.5)	-7.1	(40.8)	29.1[c]	(33.0)[d]
Medication (ADDITION) *	IT	134.1	(21.3)	180.3	(21.3)	191.3	(21.3)	200.9	(21.3)	204.1	(21.4)	182.1	(19.9)
	RC	92.8	(21.6)	124.5	(21.6)	133.1	(21.6)	133.9	(21.7)	153.6	(21.7)	127.6	(20.4)
	dif.	41.3	(30.3)	55.7	(30.3)	58.2	(30.3)	67.0	(30.4)	50.5	(30.5)	54.6[e]	(28.5)[f]
Other primary care services*	IT	398.9	(71)	412.0	(70.9)	416.7	(70.9)	503.4	(71)	539.3	(71.4)	454.1	(63.8)
	RC	352.2	(69)	348.6	(69.1)	495.8	(69.1)	505.5	(69.4)	539.7	(69.6)	448.3	(62.3)
	dif.	46.7	(98.7)	63.4	(98.9)	-79.0	(99)	-2.1	(99.3)	-0.3	(99.8)	5.7	(89.1)

# generalized linear regression models with a main effect for the intervention and for time since diagnosis and an interaction term between intervention and time; adjusted for sex and age of diagnosis and baseline HbA1c; accounted for patients being clustered in GP surgeries and observations clustered in patients

* GLM with Gaussian distribution and identity link; ** GLM Gamma distribution and log-link

Estimates used for long-term CE-model: [a] *ß total* ; [b] *SE total* ; [c] *ß consultation* ; [d] *SE consultation* ; [e] *ß medication* ; [f] *SE medication*

General note: due to the complex 3-level structure of the model estimates on single dimensions do not exactly sum up to the total cost ;
